# HLA‐B51 Positivity Correlates With Symptom Completeness From Recurrent Aphthous Stomatitis to Complete Behçet's Disease

**DOI:** 10.1111/1346-8138.17748

**Published:** 2025-04-21

**Authors:** Bo Hyun Lee, Kyung Bae Chung, Hyunwoo Jang, Yeon Woo Jung, Do‐Young Kim

**Affiliations:** ^1^ Yonsei University College of Medicine Seoul Republic of Korea; ^2^ Department of Dermatology and Cutaneous Biology Research Institute Yonsei University College of Medicine Seoul Republic of Korea

**Keywords:** Behçet's disease, clinical manifestations, gender differences, genetics, HLA‐B51

## Abstract

HLA‐B51 is well‐established as a genetic risk factor for the development of Behçet's disease (BD), and its association with certain clinical manifestations has been documented. However, there is a lack of comprehensive research examining its role in the overall symptom completeness and full clinical expression of the disease. We performed a retrospective review of medical records from 1203 patients treated at a tertiary hospital between June 2012 and June 2017. Data regarding HLA‐B51 status and clinical conditions were extracted, and patients were classified according to the “Revised Japanese Diagnostic Criteria for Behçet's disease (1987)”, resulting in 878 patients diagnosed with complete (*n* = 250) and incomplete (*n* = 628) BD. Among these patients, 400 (45.6%) were tested positive for HLA‐B51, with the positivity rate progressively increasing with the severity of symptoms. Male patients exhibited a higher likelihood of HLA‐B51 positivity (61.9%) and a greater prevalence of skin and ocular symptoms, while female patients showed a higher incidence of erythema nodosum (42.2%) and genital ulcers (91.0%). Notably, a positive correlation was observed between skin lesions and genital ulcers, whereas ocular symptoms demonstrated a negative correlation with both skin and genital manifestations. This study underscores the significant impact of HLA‐B51 positivity on the clinical presentation of BD. Insights gained from this research can enhance our understanding of genetic influences on symptom expression, ultimately leading to diagnostic and therapeutic approaches for patients with the disease.

## 1 | Introduction

Behçet's disease (BD) is a multifaceted, chronic disorder characterized by recurrent inflammation affecting multiple organ systems, with an unpredictable course and varying severity. While the exact pathogenesis remains unclear, BD is believed to result from a complex interaction of genetic, immunological, and environmental factors [1]. Among these, genetic predisposition is crucial, with the HLA‐B51 gene being the most significant marker associated with an increased risk of BD [2]. Numerous studies across different ethnic groups have confirmed a strong association between HLA‐B51 positivity and various symptoms of BD [3]. Although the degree of correlation can vary, we propose that more research is needed to clarify this variability [4]. A deeper understanding of these dynamics will enhance our insight into the gene‐phenotype interactions that influence disease progression.

This study aims to explore the connection between HLA‐B51 and symptom completeness in a large cohort of Korean BD patients. Additionally, we seek to review and compare these findings with existing data on racial differences in BD manifestations related to HLA‐B51 [5]. By integrating these results, we strive to provide a comprehensive understanding of how HLA‐B51 affects the clinical presentation of the disease.

## 2 | Methods

### 2.1 | Study Design, Patients, and Clinical Parameters

This retrospective cohort study was conducted using medical records from patients treated at the Severance Hospital, a major tertiary medical center in Seoul, South Korea. Data were collected from June 25, 2012, to June 27, 2017, to ensure a sufficient follow‐up period, allowing for comprehensive clinical evaluation and monitoring of disease progression.

Initially, we identified patients presenting with recurrent aphthous stomatitis as the primary complaint and those with a history of diagnostic evaluations for BD. Patient information, including HLA‐B51 status, age, and detailed clinical manifestations related to BD, was extracted from the hospital's electronic medical records system (SCRAP v.2.0). Selected patients were further classified based on the “Revised Japanese Diagnostic Criteria for Behçet's disease (1987)” into confirmed BD or suspected BD cases [6]. Suspected BD cases were subdivided into those presenting with oral ulcers only and those with oral ulcers accompanied by other BD‐related symptoms, such as erythema nodosum. Patients with BD‐like mucocutaneous symptoms caused by other conditions, such as inflammatory bowel disease or sarcoidosis, were excluded from the analysis. All patients included in the study had a confirmed history of at least 1 year of follow‐up or treatment. Cases with insufficient follow‐up or visits, leading to incomplete diagnostic evaluations, were excluded from the analysis.

### 2.2 | Symptom Completeness Evaluation

For diagnostic evaluation, we applied the “Revised Japanese Diagnostic Criteria for Behçet's Disease (1987)” [6], which divides patients based on the presence of major and minor symptoms. Major ones include oral ulcers, skin lesions, genital ulcers, and ocular symptoms, while minor symptoms encompass arthritis, epididymitis, gastrointestinal lesions, vascular involvement, and central nervous system (CNS) symptoms. Based on the number and combination of these symptoms, patients were classified as complete, incomplete, or suspected cases. To refine the analysis, suspected BD was further stratified into two subcategories: patients presenting with oral ulcers only and those with oral ulcers accompanied by a BD‐related extraoral symptom (e.g., erythema nodosum). This stratification allowed the concept of “symptom completeness” to be redefined into four stages, reflecting how comprehensively the most prevalent symptoms are represented and suggesting a progression from oral ulcers alone to complete BD. Additionally, for complementary verification, we incorporated the International Criteria for Behçet's Disease (ICBD) by linearly converting its diagnostic threshold of 4 points or more into a symptom completeness scale, providing further supporting evidence for the analysis of clinical progression [7].

### 2.3 | Statistical Analysis

Odds ratios were calculated for each parameter using 2 × 2 contingency tables with 95% confidence intervals. A Student's *t*‐test was employed to assess differences between groups, with statistical significance set at *p* less than 0.05. Significant values are highlighted in bold within the results tables.

R version 4.4.1 was used for data visualization, including bar graphs and heatmaps. The correlation between two binary parameters was determined using the Phi coefficient, and the results were visualized using heatmaps.

## 3 | Results

**FIGURE 1:**
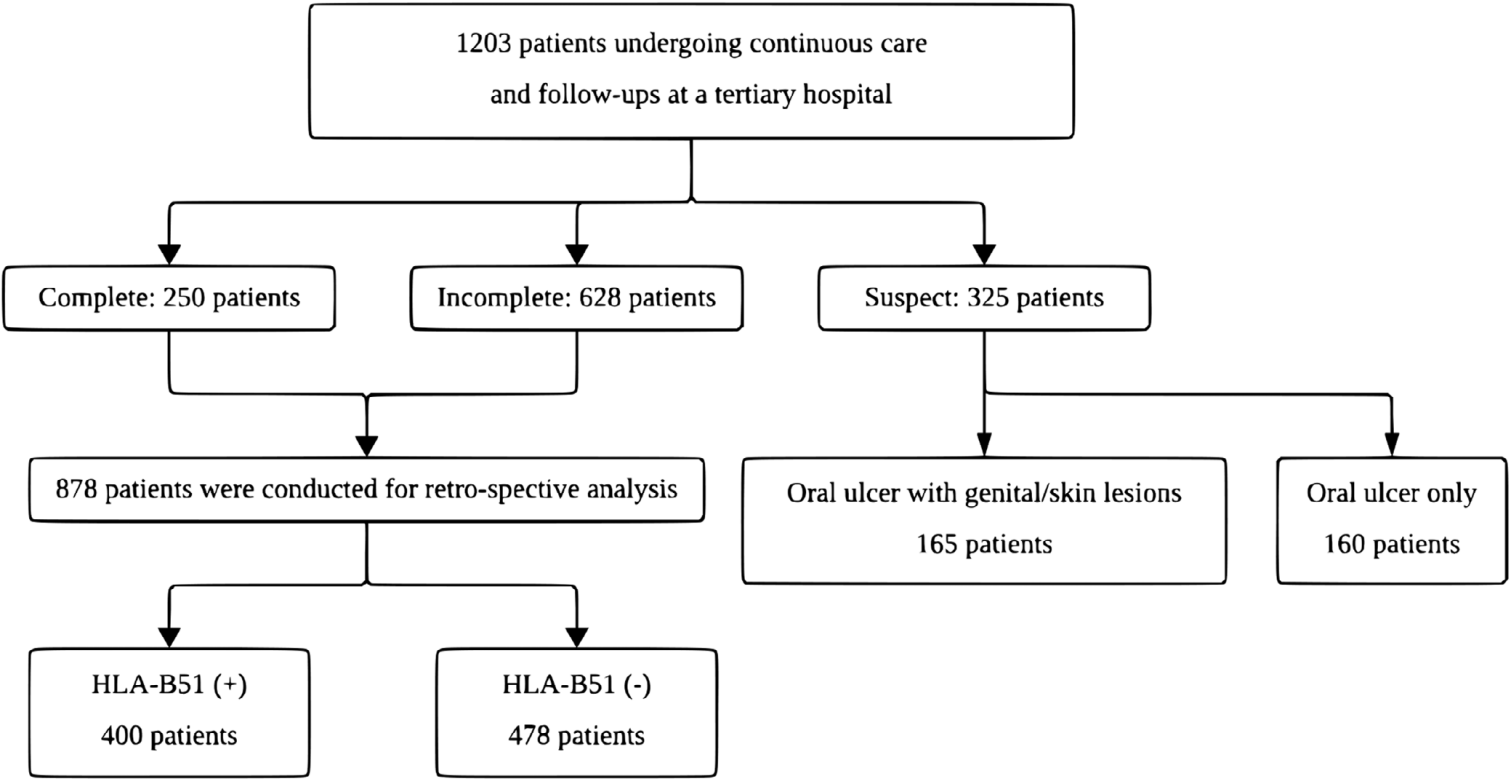
Flowchart of participant classification and analysis.

The flowchart depicting the study participants is illustrated in Figure 1. Among the 1203 patients who presented with oral ulcers and had their HLA‐B51 status pre‐assessed, complete (*n* = 250), incomplete (*n* = 628), and suspect (*n* = 325) were classified according to the Japanese Criteria. Since both complete and incomplete cases meet the BD diagnostic criteria, 878 patients were included in the analysis. Of these, 400 (45.6%) were HLA‐B51 positive, while 478 (54.4%) were HLA‐B51 negative. Among the remaining 325 patients classified as suspected, all had oral ulcers. Additionally, 165 of these exhibited genital ulcers or skin lesions, while the remaining 160 had only oral ulcers.

**FIGURE 2:**
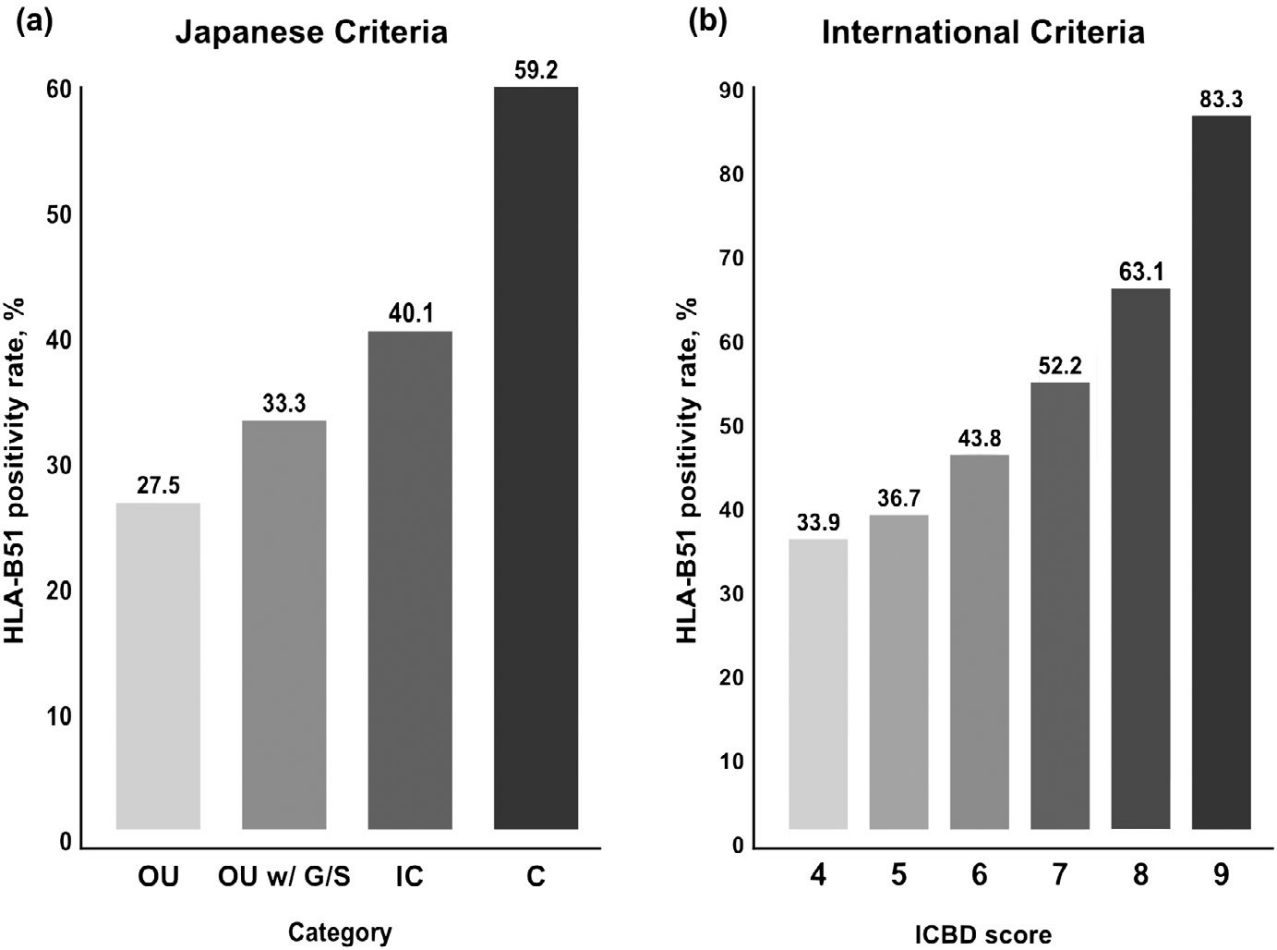
Association between increasing symptom completeness defined by each method and HLA‐B51 positivity rate. (a) Based on the Japanese Criteria, the oral ulcer (OU), oral ulcer with genital/skin lesions (OU w/G/S), incomplete (IC), and complete (C) symptom groups are shown. (b) Based on the International Criteria for Behçet's Disease.

The distribution of patients across different groups according to gender and HLA‐B51 status is presented in Table S1. As symptom completeness increased from the ‘only oral ulcers’ group to the “complete” group, the HLA‐B51 positivity rate rose sequentially from 27.5%, 33.3%, 40.1% to 59.2%. This trend is illustrated in Figure 2a. Moreover, even when using the ICBD criteria, the HLA‐B51 positivity rate shows a gradual increase among patients with a score of 4 or higher, who can be diagnosed as BD in Figure 2b. The classification of the 878 study participants according to major and minor criteria is detailed in Table S2.

Tables 1 and 2 present the statistical analysis of clinical symptoms by gender and HLA‐B51 status, while Table 3 provides the corresponding odds ratios (ORs) of the major criteria. Males exhibited a higher likelihood of being HLA‐B51 positive (61.9% vs. 40.4%) and showed an increased prevalence of papulopustular lesions (63.3% vs. 52.1%, OR: 1.588), ocular involvement (57.6% vs. 32.9%, OR: 2.769), CNS involvement (14.8% vs. 6.3%, OR: 2.581), and vascular involvement (15.7% vs. 4.8%, OR: 3.706). Notably, the odds of ocular involvement in males were 2.77 times higher than in females. In contrast, erythema nodosum (42.2% vs. 33.3%, OR: 0.684) and genital involvement (91.0% vs. 73.3%, OR: 0.271) were significantly more common in females, indicating a 1.462‐fold and 3.69‐fold higher prevalence, respectively.

**TABLE 1:** Distribution of clinical symptoms in complete and incomplete Behçet's disease patients by gender.

Parameters	Patients (*n* = 878)	Gender		*p*
		Male (*n* = 210)	Female (*n* = 668)	
Age at observation, years, mean ± SD	51.3 ± 11.3	49.6 ± 11.9	51.8 ± 11.0	—
HLA‐B51 positive	400 (45.6)	130 (61.9)	270 (40.4)	< 0.001
Skin involvement	820 (93.4)	200 (95.2)	620 (92.8)	0.266
Papulopustular lesions	481 (54.8)	133 (63.3)	348 (52.1)	0.005
Erythema nodosum	352 (40.1)	70 (33.3)	282 (42.2)	0.024
Others^a^	49 (5.6)	16 (7.6)	33 (4.9)	0.167
Oral involvement	878 (100.0)	210 (100.0)	668 (100.0)	1.000
Ocular involvement	341 (38.8)	121 (57.6)	220 (32.9)	< 0.001
Genital involvement	762 (86.8)	154 (73.3)	608 (91.0)	< 0.001
Pathergy reaction	430 (49.0)	101 (48.1)	329 (49.3)	0.813
CNS involvement	73 (8.3)	31 (14.8)	42 (6.3)	< 0.001
Gastrointestinal involvement	230 (26.2)	62 (29.5)	168 (25.1)	0.209
Joint involvement (Arthritis)	327 (37.2)	85 (40.5)	242 (36.2)	0.288
Vascular involvement	65 (7.4)	33 (15.7)	32 (4.8)	< 0.001

Abbreviation: SD, standard deviation.

^a^ Others include superficial thrombophlebitis, as well as potentially other dermatological conditions associated with the disease, like Sweet syndrome.

**TABLE 2:** Distribution of clinical symptoms in complete and incomplete Behçet's disease patients by HLA‐B51 status.

Parameters	HLA‐B51 positive (*n* = 400)	HLA‐B51 negative (*n* = 478)	*p*
Age at observation, years, mean ± SD	51.3 ± 11.0	51.3 ± 11.5	—
Gender, Male	130 (32.5)	80 (16.7)	< 0.001
Skin involvement	384 (96.0)	436 (91.2)	0.006
Papulopustular lesions	233 (58.3)	248 (51.9)	0.066
Erythema nodosum	179 (44.8)	173 (36.2)	0.011
Others^a^	23 (5.8)	26 (5.4)	0.883
Oral involvement	400 (100.0)	478 (100.0)	1.000
Ocular involvement	170 (42.5)	171 (35.8)	0.044
Genital involvement	359 (89.8)	403 (84.3)	0.021
Pathergy reaction	197 (49.3)	233 (48.7)	0.892
CNS involvement	58 (14.5)	15 (3.1)	< 0.001
Gastrointestinal involvement	86 (21.5)	144 (30.1)	0.004
Joint involvement (Arthritis)	144 (36.0)	183 (38.3)	0.528
Vascular involvement	48 (12.0)	17 (3.6)	< 0.001

Abbreviations: CNS, central nervous system; SD, standard deviation.

^a^ Others include superficial thrombophlebitis, as well as potentially other dermatological conditions associated with the disease, like folliculitis.

**TABLE 3:** Evaluation of associations between core symptoms, gender and HLA‐B51 status.

OR (95% CI)	Oral involvement	Ocular involvement	Genital involvement	Skin involvement	Papulopustular lesions	Erythema nodosum
Male gender	—	2.769*** (2.015–3.804)	0.271*** (0.181–0.407)	1.548 (0.769–3.117)	1.588** (1.154–2.185)	0.684* (0.494–0.948)
HLA‐B51 positivity	—	1.327* (1.01–1.743)	1.63* (1.085–2.447)	2.312** (1.279–4.179)	1.294 (0.99–1.691)	1.428** (1.089–1.873)

*Note:* *p < 0.05, **p < 0.01, ***p < 0.001.

Abbreviations: CI, confidence interval; OR, odds ratio.

HLA‐B51 positivity was associated with increased odds of multiple clinical manifestations. Patients carrying the HLA‐B51 allele had a higher prevalence of erythema nodosum (44.8% vs. 36.2%, OR: 1.428), ocular involvement (42.5% vs. 35.8%, OR: 1.327), genital involvement (89.8% vs. 84.3%, OR: 1.63), CNS involvement (14.5% vs. 3.1%, OR: 5.235), and vascular involvement (12.0% vs. 3.6%, OR: 3.698). Conversely, HLA‐B51 positivity appeared to decrease the likelihood of gastrointestinal involvement (21.5% vs. 30.1%, OR: 0.635), suggesting a potential protective effect in this subset.

**FIGURE 3:**
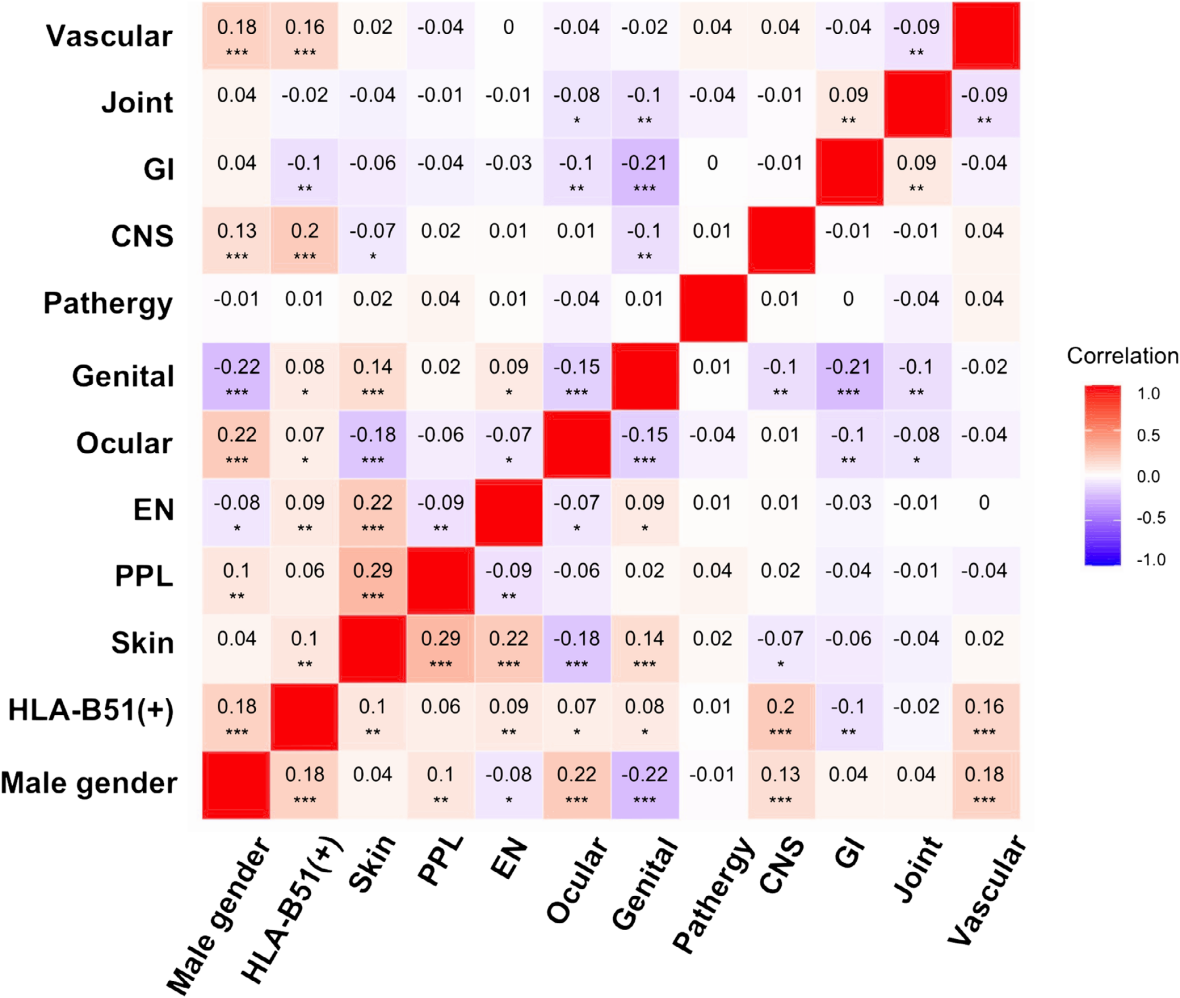
Heatmap that shows correlation between clinical factors in total patients. CNS, central nervous system; EN, erythema nodosum; GI, gastrointestinal; PPL, papulopustular lesion.

The analysis of correlations between clinical symptoms within the groups of patients diagnosed with BD is shown in Figure 3. Beyond the previously discussed associations with male gender and HLA‐B51 positivity, additional noteworthy correlations are observed, with coefficients exceeding 0.1. As expected, papulopustular lesions and erythema nodosum showed strong positive correlations with overall skin involvement (*r* = 0.29 and *r* = 0.22, respectively) and were also closely interrelated, indicating that these two skin manifestations are not mutually independent but rather frequently co‐occurring features of BD. Additionally, a moderate positive correlation was observed between skin lesions and genital ulcers (*r* = 0.14), suggesting a tendency for these mucocutaneous symptoms to present together. Furthermore, CNS lesions showed a positive correlation with male gender (*r* = 0.13) and HLA‐B51 positivity (*r* = 0.2), while vascular lesions exhibited positive correlations with male gender (*r* = 0.18) and HLA‐B51 positivity (*r* = 0.16). In contrast, negative correlations were found between skin lesions and ocular symptoms (*r* = −0.18), genital ulcers and ocular symptoms (*r* = −0.15), as well as genital ulcers and gastrointestinal symptoms (*r* = −0.21). These findings suggest that while skin and genital manifestations tend to co‐occur, ocular and gastrointestinal symptoms may present in a mutually exclusive manner.

## 4 | Discussion

This study highlights the relationship between HLA‐B51 positivity and symptom completeness in BD. Previous research has shown that HLA‐B51 positivity significantly increases the likelihood of various symptoms [8, 9]. Our findings align with this, as we found that patients with HLA‐B51 positivity are more likely to experience issues such as erythema nodosum, genital ulcers, and ocular symptoms, while gastrointestinal symptoms were less common. These patterns are consistent with prior studies [10, 11]. Additionally, CNS involvement has been reported to correlate positively with HLA‐B51 positivity [12] and male gender [13], while vascular lesions have also been shown to be associated with HLA‐B51 positivity [14] and male gender [15]. Particularly, when classifying confirmed BD cases using the Japanese criteria, the allele frequency of HLA‐B51 is notably higher in complete‐type BD than in incomplete‐type BD. This pattern has also been observed in previous Japanese studies, supporting the idea that HLA‐B51‐associated ocular manifestations play a crucial role in the classification of complete BD [16].

Males were more likely to present papulopustular lesions and ocular symptoms, while females showed a higher prevalence of erythema nodosum and genital ulcers in our analysis. These gender‐based differences have been consistently observed across various ethnic cohorts, including Turkish, Iranian, and East Asian populations, where males exhibit a higher prevalence of acneiform pustular lesions and females more frequently develop erythema nodosum [17, 18, 19, 20]. However, in our analysis, the connection between papulopustular lesions and HLA‐B51 positivity didn't reach statistical significance. Similar challenges in finding significant relationships have been reported [12, 21].

When exploring the relationships between major symptoms, we noted some interesting negative correlations, especially between ocular symptoms and skin lesions or genital ulcers. In the group of patients with incomplete symptoms, many lacked ocular involvement, which likely affected these relationships. This suggests potential issues in symptom categorization, especially according to the Japanese Criteria.

Several limitations of our study should be acknowledged. First, we found a lower prevalence of ocular symptoms (38.8%) compared to the usual range of 45%–90%. This discrepancy may reflect specific characteristics of our patient population, like findings in other studies [15, 22]. Our retrospective data collection didn't account for the age of onset of symptoms, limiting our analysis to their presence over 5 years. Previous research emphasizes the importance of this factor in understanding symptom progression [22, 23]. Incorporating this information could provide valuable insights into how genetic factors influence the duration and patterns of symptoms in BD. Additionally, the lack of detailed analysis of the phenotype of HLA‐B51‐negative individuals is a notable limitation. Studies should include this subgroup to better understand the phenotypic variations in BD.

This study confirms that while both genetic and environmental factors contribute to BD, the genetic component, particularly HLA‐B51 positivity, exerts a significant influence on its clinical manifestations. By analyzing the largest cohort in a single South Korean institution, we provided a comprehensive overview of the correlations between clinical presentations, gender, and HLA‐B51 positivity. Our findings demonstrate that these genetic factors notably affect the major criteria, leading to an increase in symptom completeness, which in turn correlates with a linear rise in gene positivity rate. Furthermore, our results align closely with findings from similarly structured Japanese studies, highlighting regional consistency in how HLA‐B51 positivity impacts the clinical manifestations of BD, as observed in this large Korean cohort. While we focused on quantifying the effect of HLA‐B51 positivity on disease progression in a Korean cohort, further studies should explore how regional and ethnic differences shape the prevalence and clinical spectrum of BD. Expanding this analysis across diverse populations will provide deeper insights into the interplay between genetic predisposition and symptom severity, further refining our understanding of BD pathophysiology.

## Ethics Statement

This retrospective study was approved by the Institutional Review Board at Severance Hospital, Seoul, Korea (approval # 4‐2021‐0724).

## Conflicts of Interest

The authors declare no conflicts of interest.

## Supporting information


Tables S1–S2.
