# Supplementary material for: HLA‐B51 Positivity Correlates With Symptom Completeness From Recurrent Aphthous Stomatitis to Complete Behçet's Disease
Source: J Dermatol. 2025 Apr 21;52(6):1001–7. doi: 10.1111/1346-8138.17748 (PMC12149367; doi:10.1111/1346-8138.17748)
Supplement: Supplementary file 1 — Tables S1–S2. [file JDE-52-1001-s001.docx]

**SUPPLEMENTARY TABLE 1** Overview of patient classification by Japanese criteria

| **Japanese Criteria** | **Gender†** | | **HLA-B51 status†** | | | **Total**  **(n=1203)** |
| --- | --- | --- | --- | --- | --- | --- |
|  | **Male**  **(n=313)** | **Female**  **(n=890)** | | **HLA-B51 positive**  **(n=499)** | **HLA-B51 negative**  **(n=704)** |  |
| Complete | 92(36.8) | 158(63.2) | | 148(59.2) | 102(40.8) | 250 |
| Incomplete | 118(18.8) | 510(81.2) | | 252(40.1) | 376(59.9) | 628 |
| Oral ulcer with genital/skin lesions | 41(24.8) | 124(75.2) | | 55(33.3) | 110(66.7) | 165 |
| Oral ulcer only | 62(38.8) | 98(61.3) | | 44(27.5) | 116(72.5) | 160 |

**†**: () is ratio of each criteria count

**SUPPLEMENTARY TABLE 2** Patient distribution based on major and minor symptoms

| **Japanese Criteria** | **Major symptom** | | | | **Minor symptom†** | **Count** |
| --- | --- | --- | --- | --- | --- | --- |
|  | **Oral** | **Ocular** | **Genital** | **Skin** |  |  |
| Complete | O | O | O | O | - | 250 |
| Incomplete | O | O | O | X | - | 24 |
|  | O | O | X | O | - | 49 |
|  | O | X | O | O | - | 472 |
|  | O | O | X | X | - | 18 |
|  | O | X | O | X | 2 minors | 16 |
|  | O | X | X | O | 2 minors | 49 |

**†**: Minor symptoms include arthritis, epididymitis, gastrointestinal, vascular lesions, central nervous system (CNS)
